# Oral transmucosal fentanyl citrate analgesia in prehospital trauma care: an observational cohort study

**DOI:** 10.1186/s13049-023-01066-0

**Published:** 2023-01-07

**Authors:** Urs Pietsch, Henning Fischer, Christoph Alexander Rüst, Björn Hossfeld, Andreas Grünenfelder, Volker Wenzel, Roland Albrecht

**Affiliations:** 1 Department of Anesthesiology and Intensive Care Medicine, Cantonal Hospital St. Gallen, Rorschacher Strasse 95, 9007 St. Gallen, Switzerland; 2 Swiss Air-Ambulance, Rega (Rettungsflugwacht/Guarde Aérienne), Zurich, Switzerland; 3 Department of Emergency Medicine, Inselspital, Bern University Hospital, University of Bern, Bern, Switzerland; 4 Department of Intensive Care Medicine, Cantonal Hospital Frauenfeld, Frauenfeld, Switzerland; 5 Federal Armed Forces Hospital, Department of Anesthesiology, Intensive Care Medicine, Emergency Medicine and Pain Therapy, and HEMS, Christoph 22” Ulm, Ulm, Germany; 6 Department of Anesthesiology, Klinik Gut, St. Moritz, Switzerland; 7 Department of Anaesthesiology and Intensive Care Medicine, Friedrichshafen Regional Hospital, Friedrichshafen, Germany; 8 Department of Anesthesiology, University of Florida, Gainesville, FL USA

**Keywords:** OTFC, Fentanyl, Prehospital analgesia, Trauma support, HEMS

## Abstract

**Background:**

Pain is one of the major prehospital symptoms in trauma patients and requires prompt management. Recent studies have reported insufficient analgesia after prehospital treatment in up to 43% of trauma patients, leaving significant room for improvement. Good evidence exists for prehospital use of oral transmucosal fentanyl citrate (OTFC) in the military setting. We hypothesized that the use of OTFC for trauma patients in remote and challenging environment is feasible, efficient, safe, and might be an alternative to nasal and intravenous applications.

**Methods:**

This observational cohort study examined 177 patients who were treated with oral transmucosal fentanyl citrate by EMS providers in three ski and bike resorts in Switzerland. All EMS providers had previously been trained in administration of the drug and handling of potential adverse events.

**Results:**

OTFC caused a statistically significant and clinically relevant decrease in the level of pain by a median of 3 (IQR 2 to 4) in NRS units (*P* < 0.0001). Multiple linear regression analysis showed a significant absolute reduction in pain, with no differences in all age groups and between genders. No major adverse events were observed.

**Conclusions:**

Prehospital administration of OTFC is safe, easy, and efficient for extrication and transport across all age groups, gender, and types of injuries in alpine environments. Side effects were few and mild. This could provide a valuable alternative in trauma patients with severe pain, without the delay of inserting an intravenous line, especially in remote areas, where fast action and easy administration are important.

## Introduction

Pain is one of the major prehospital symptoms in trauma patients and requires prompt management. Early pain treatment following injury can improve long-term outcomes, while untreated pain can facilitate higher post-traumatic stress disorder (PTSD) rates and worsen outcomes [1]. Nevertheless, recent data have recognized that insufficient analgesia after prehospital treatment by emergency medical services (EMS) is reported to be as high as 43%, and is therefore a significant area for improvement [2].

There are various reasons for this failure, such as a lack of adequately skilled rescuers and long EMS response times due to mountainous terrain in remote areas. In Switzerland, with outdoor activities such as skiing and mountain biking resulting in > 76,000 injured patients per year, the need for sufficient prehospital analgesia is considerable [3–5]. Inadequate analgesia in this prehospital environment can be compounded by additional challenges such as extremely cold temperatures, wind, logistical constraints, evacuation and monitoring limitations, and more demanding intravenous access imposed by the hostile alpine environment [5]. Novel pain management strategies must address these issues in this special prehospital setting.

Use of an oral lozenge containing transmucosal fentanyl citrate (OTFC) has been identified as a possible strategy to address this need and has been used by the military since the 1990s, predominantly within the U.S. special operations community [6, 7]. In addition, the OTFC lozenge has recently been adopted by the UK Ministry of Defence as the primary method for immediate analgesia on the battlefield [7]. Implementation in civilian practice of clinical innovations from recent military campaigns is essential to ensure that hard-won lessons are translated for use in a civilian application [8, 9]. Unfortunately, there is limited data describing the use of OTFC for civil prehospital trauma patients so far [10]. Therefore, our goal was to apply this experience in a similar setting, namely mountain rescue in the Swiss alps, where extrication requires simple usage and fast acting pain relief that can be safely administered by first responders and EMS personnel without the need for an intravenous line. In an observational cohort study, prehospital pain reduction and side effects were recorded. We hypothesized that the use of OTFC for trauma patients in this remote and challenging environment is feasible, efficient, and safe, and might be an alternative to nasal and intravenous applications.

## Methods

This observational cohort study examined patients who were treated with OTFC from July 2020 to May 2022. Reporting of the study conforms with the STROBE statement for the reporting of observational cohort studies. The local ethics committee evaluated and approved the study and waived written informed consent (BASEC Nr. 2020–00,096 EKOS 21/006).

### Study design and participants

All patients with severe pain, defined as a score of ≥ 4 on a numeric rating scale (NRS), with 0 defined as absence of pain and 10 being the maximum imaginable pain, who did not have i.v. access were included. If there was evidence of altered consciousness, alcohol consumption or noticeable abnormal vital signs, administration of OTFC was deemed to be contraindicated. Head trauma (GCS < 15) was an absolute contraindication for OTFC. Further OTFC contraindications were known allergy to the drug or its additives, patient refusal, or age < 14 years. Major adverse events were defined as apnea and severe bradypnea requiring ventilation as an expression of overdosing.

All EMS providers, including ski patrol rescuers, EMS paramedics, and prehospital emergency physicians, received theoretical and practical instruction about OTFC. Specifically, they were instructed about the mechanisms of action, indications and contraindications, as well as potential side effects. The study group consisted of trauma patients predominantly participating in recreational sport in the Ski and Bike resort Lenzerheide and St. Moritz (Switzerland), Ski World Cup (FIS Ski Worldcup St. Moritz, Lenzerheide, Switzerland), Mountainbike World cup (UCI World Cup Lenzerheide Downhill and Cross Country, Switzerland), Bike Park and MTB Stage Races (Swiss Epic, Bike Giro Engadin, Switzerland).

### Medication

The medication used is OTFC 600/800 mcg (Actiq, Teva Pharma AG, Basel, Switzerland). The single dose was 600 mcg oral fentanyl for the first 65 patients. This amount was later changed to 800 mcg.

The original dose of 600 mcg was chosen according to the buccal absorption equivalent of about 20% of intravenous fentanyl. When employing this route of medication, the onset is nearly as fast as that of intravenous fentanyl, as there is no hepatic first pass effect.

Because of limited availability, the dose was changed to 800 mcg lozenges. There is hardly any uptake of the drug via the intestines [13]. Each EMS member participating in the study was additionally carrying a dose of 1.8 mg naloxone (Nyxoid, Mundipharma Medical Company, Basel, Switzerland) for nasal application as an antidote. Additional medication after extrication was only administered if it was indicated and a physician was on scene, which was predominantly the case in HEMS deployment.

### Data collection and statistical analysis

Data collection was performed by the medical provider using an app-based online protocol with predefined endpoints, prospectively recording the data in Table 1 (except retrospective HEMS data). The patient’s and mission’s characteristics were examined, including vital signs (heart rate, oxygen saturation, AVPU scale, GCS and NRS) on initial presentation to the prehospital team and upon admission to the emergency department (mechanism of injury, type of transport, side effects and need for additional drugs). The overall safety profile for OTFC was determined by the incidence of adverse events, defined as interrupted use, nausea, drowsiness, or respiratory depression. No further personal data were recorded in order to ensure de-identification of patients. Also, no personal data of the first responders on scene were collected, so no conclusions about the rescuers could be drawn during later analysis. Additionally, the HEMS and EMS charts were analysed retrospectively in relation to further analgesia requirements or side effects during transport to the hospital (Table 1).

**Fig. 1:**
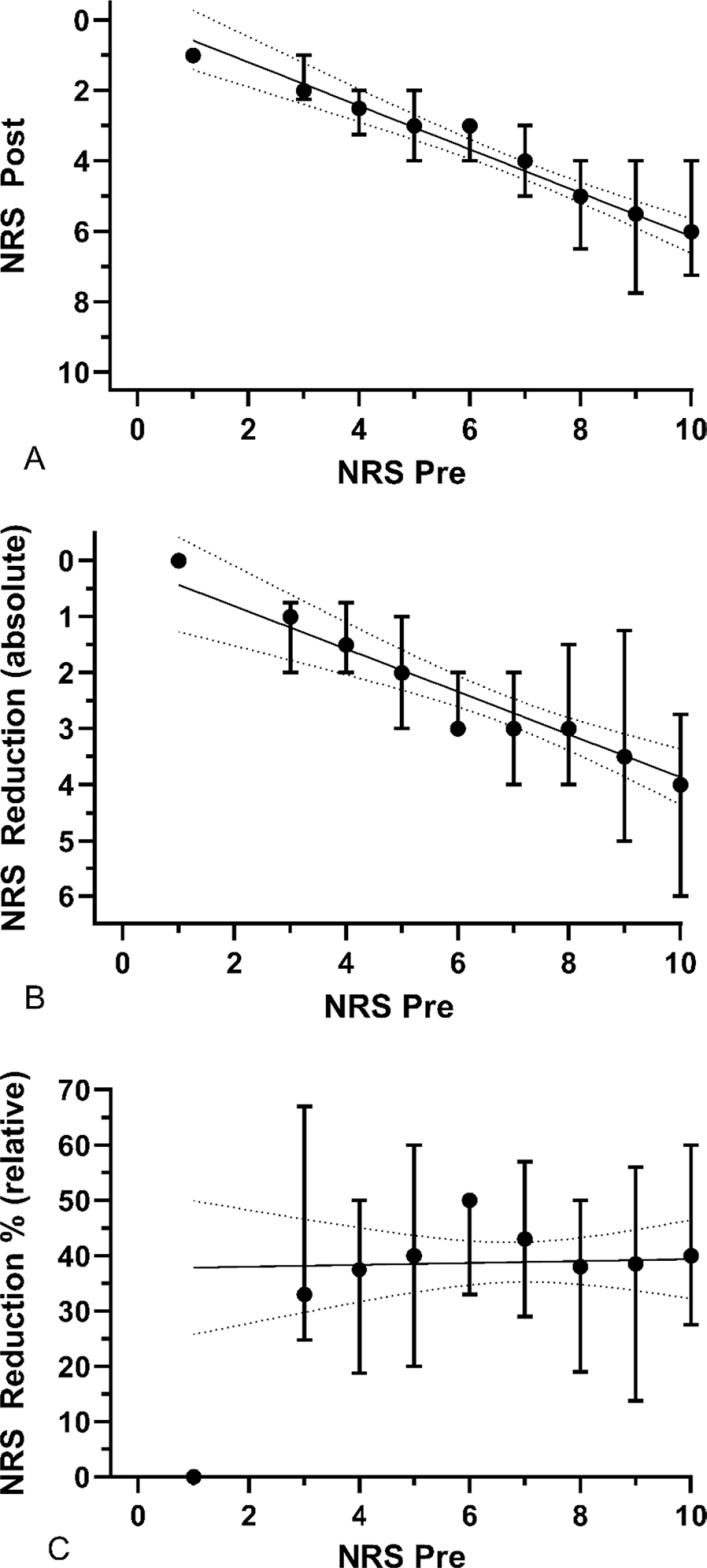
Distribution of absolute and relative reduction depending on pain level. Footnote: Graph A shows the pain level before and after administering OTFC. Graph B includes the absolute reduction depending on pain level before OTFC. Moreover, the effect of higher efficiency with higher NRS when administering fentanyl can be seen in both. Graph C includes the relative reduction in percentage. Here, we were able to show equal pain reduction depending on the initial pain level, with an average just below 40%

**Table 1:** Baseline characteristics

Variable	Total n = 177
*Age in years*	
Mean, ± SD	40 ± 17
< 20, n (%)	25 (14.1)
20–60, n (%)	130 (73.5)
≥ 60, n (%)	22 (12.4)
Male gender, n (%)	106 (59.9)
*Pain (NRS)*	
Initial, median (IQR)	7 (6 to 8)
After OTFC	4 (3 to 5)
Mean pain reduction	3 (2 to 4)
*Dose of OTFC*	
600 µg, n (%)	65 (36.7)
800 µg, n (%)	112 (63.3)
*Location of Trauma*	
Upper extremity, n (%)	90 (50.8)
Lower extremity, n (%)	81 (45.8)
Thorax, n (%)	5 (2.8)
Abdomen, n (%)	2 (1.1)
Spinal column, n (%)	3 (1.7)
*Adverse Events*	
None, n (%)	159 (89.8)
Nausea/Vomiting, n (%)	8 (4.5)
Vertigo, n (%)	10 (5.6)
Bradypnea, n (%)	1 (0.5)
Tachypnea, n (%)	3 (1.6)
Major adverse events, n (%)	0 (0)
*Transport mode to Hospital*	
Ground EMS, n (%)	137 (77.4)
Helicopter EMS, n (%)	40 (22.6)
*Helicopter EMS treatment*	
Additional i.v. line, n (%)	26 (14)
Additional i.v. medication, n (%)	14 (53.9)
Fentanyl, Ketamine, midazolam, n (%)	2 (14.3)
Ketamine, Midazolam, n (%)	3 (21.3)
Ketamine, Fentanyl, n (%)	2 (14.3)
Fentanyl, n (%)	5 (35.7)
Ketamine, n (%)	2 (14.3)

No missing data

*SD* Standard Deviation, *IQR* Inter-Quartile Range, *OTFC* Oral Transmucosal fentanyl citrate

### Statistical analysis

Patient characteristics are summarized and presented in tables. Continuous variables were summarized by mean ± standard deviation if normally distributed, or by median and the interquartile range if skewed. To increase the quality of data analysis, each set of data was tested for normal distribution (D’Agostino and Pearson omnibus normality test) and for homogeneity of variance (Levene’s test) before statistical analyses. To find differences between paired groups (e.g., NRS before and after administration of fentanyl), a paired *t*-test was used in case of normally distributed data, and a Wilcoxon matched-pairs signed rank test was used in case of not normally distributed data. To find differences between two unpaired groups, Student’s *t*-test was used in case of normally distributed data (with Welch’s correction in case of unequal variance), and a Mann–Whitney test was used in case data were not normally distributed. To find differences between multiple unpaired groups (e.g., reduction in NRS after application of fentanyl in different age groups), a one-way ANOVA and subsequent Tukey–Kramer multiple comparisons post-hoc test with a single pooled variance was used in case of homoscedastic data with normally distributed residuals. In case of heteroscedastic data with normally distributed residuals, a Brown-Forsythe and Welch ANOVA with subsequent Dunnett’s T3 multiple comparisons post-hoc test, and with individual variances computed for each comparison (n > 50 / group) was used. In case of not normally distributed residuals, a Kruskal–Wallis test with a subsequent Dunn’s multiple comparisons test was applied. To show the connection between pre-therapeutics NRS and effectiveness of therapy, simple linear regression analysis was used. Furthermore, a multiple linear regression with NRS-reduction as dependent and gender, age group and initial NRS as main effects was calculated, whereas male gender as well as the age group < 20 years old was used as reference. Statistical analyses were performed using IBM SPSS Statistics (Version 24.0.01, IBM SPSS, Chicago, IL, USA) and GraphPad Prism (version 9.4.0 for Windows, GraphPad Software, San Diego, CA, USA). Significance was accepted at *P* < 0.05 (two-sided for *t*-tests and exact for Mann–Whitney tests).

## Results

During the observation period, a total of 177 patients (106 male sex, 59.9%; age, 40 ± 17 years) were treated with OTFC (Table 1). The most common body region being injured were the upper extremities in 90 patients (50.8%), followed by the lower extremities with 81 injuries (45.8%). The remaining 10 (5.6%) were injuries of the spinal column, thorax and abdomen. Due to the low case load, we included these patients in one common subgroup (Table 1). Some patients suffered multiple injuries. The trauma victims reported a median pain level of 7 (IQR 6 to 8) on the numeric rating scale during initial assessment by an EMS provider. OTFC caused an overall statistically significant and clinically relevant decrease in the level of pain by a median of 3 (IQR 2 to 4) in NRS units (*P* < 0.0001) for the main subgroups, while in the thorax, abdomen and spine, subgroup pain reduction was 3 (IQR 2 to 5) in NRS units (*P* < 0.001).

Further inter-group analyses of pain relief between sexes, age groups and locations of injury showed that the effect of OTFC was not different between each set of groups, regardless of whether absolute values of pain reduction (in NRS) or relative values (expressed in % of initial NRS) were evaluated (Table 2).

**Table 2:** Pain reduction in different subgroups

Subgroup	NRS initial, n (IQR)	NRS after OTFC, n (IQR)	NRS Reduction absolute, n (IQR)	Pain Reduction Percentage (SD)	*P* value
Men	7 (6–8)	4 (3–6)	3 (2–4)	39.8% (24.6%)	*P* < 0.0001
Women	7 (5–8)	4 (3–5)	2 (1–3)	37.4% (23.6%)	*P* < 0.0001
Age < 20	7 (6–8)	4 (3–5)	3 (2–4)	43.8% (26.9%)	*P* < 0.0001
Age 20–60	7 (5.25–8)	4 (3–5.75)	2 (1–4)	38.1% (23%)	*P* < 0.0001
Age > 60	7 (5.75–9)	5 (3–7.25)	2.5 (1–4)	34.5% (26.6%)	*P* < 0.0001
Upper Extremities	7 (6–8)	4 (3–6)	2 (1.75–4)	36.7% (22.4%)	*P* < 0.0001
Lower Extremities	7 (5–8)	4 (2–5)	3 (1–4)	39.8% (26.2%)	*P* < 0.0001
Thorax, Abdomen and Spine	7 (6–8)	3 (3–5.5)	3 (2–5)	45.7% (25%)	*P* < 0.001

Absolute pain reduction in all subgroups was around 40%, showing an efficient analgetic effect of OTFC on a broad range of patients. The subgroups of injuries in the thorax, abdomen and the spinal column combined due to small case load each. Additionally, we could show that absolute reduction in pain (expressed in NRS) was directly proportional to the initial pain level (see Fig. 1, Graph A to C), whereas the relative reduction in pain (expressed in % of initial NRS) was stable throughout the initial intensity of pain

*IQR* inter-quartile range, *SD* standard deviation, *P* significant < 0.05

Multiple linear regression analysis showed that absolute reduction in pain was only dependent of initial pain but independent from sex or age group. Out of 177 patients, 137 (77.4%) were transported by GEMS and thus did not receive any further anaesthetics; 40 (22.7%, 31 males, 9 female) were transported by HEMS. Twenty-six of these patients (14%) received an i.v.-line with further analgesia and sedation after hand-over to a HEMS physician based on the severity of the injuries (predominantly long bone fractures, in one case open, and shoulder reduction). The OTFC was well tolerated overall, with only a few mild side effects such as vertigo in 10 (5.6%) and/or nausea in 8 (4.5%), out of a total of 15 patients (8.4% of 177). Breathing remained unaffected in 173 patients (97.9%), with one case of mild bradypnea (0.5%) and 3 cases of tachypnea (1.6%). No major adverse events occurred, such as overdosing that would have to be reversed by administration of the carried naloxone or supported by BVM ventilation.

## Discussion

We found OTFC to be an effective, safe and feasible strategy for pain relief in civilians treated in remote prehospital environments. Following thorough instruction, lozenges were successfully administered by EMS personnel without any major adverse events. The NRS scale was chosen as being the easiest and most well-established pain score used over the past years by our rescue personnel. In triage algorithms and treatment guidelines, pain scores are used to determine treatment priority and pain management. The verbal rating scale (VRS) or numerical rating scale (NRS) are such validated quick and easy pain scores for measuring pain and are commonly used to quantify subjective pain perception. The observed reduction in pain of 3 (IQR 2 to 4) NRS points was equal or even superior to other analgesia strategies in remote terrain—such as methoxyflurane with a 2.9-point NRS reduction—and is consistent with the findings for OTFC in a military setting [5, 10–12]. In the literature, pain reduction of more than 25% is generally seen as sufficient treatment of pain; with a value of about 40% in each of our subgroups, we consider the effect successful. Additionally, we were able to show that there were no differences in efficiency in gender, a wider variety of ages, or in the body region of injury.

Although there are different ways of successfully distributing strong analgesia without establishing an i.v. line, such as nasal administration of fentanyl, ketamine or nalbuphine [3, 10, 12], a potential downside of nasal administration can be slower onset, especially in cold environments, and a weaker effect due to vasoconstriction of the nasal vessels or congestion of the nasal cavity with mucus. Enteral application tends to be too slow in onset and does not play a significant role in the time-dependent prehospital setting. Moreover, medical gases for analgesia, such as methoxyflurane, require carrying of relatively bulky equipment such as gas canisters.

In a hostile alpine environment, surrounding conditions can play a significant role in keeping the prehospital scene time as short as possible. The OTFC lozenge can be given to patients as soon as the EMS team is on the scene, no matter whether the team member is a first responder, paramedic or physician, and allows prehospital teams to deliver rapidly effective, potent analgesia without requiring i.v. access. This is a significant time advantage in dangerous, resource-limiting environments such as remote alpine slopes, where there may be cold, snow and risk of avalanches or falling rocks. Using effective, non-i.v.-line-dependent analgesia may contribute to even faster extraction without further delay.

Delivering fentanyl in a solid state significantly reduces the risk of ampule breakage and thus injuries. There is no freezing at very low temperatures, and it is ready to use almost immediately. In our trauma cohort, the lozenges were predominantly used in winter sport accidents, where reaching these patients and establishing early i.v. access can be challenging due to the harsh environmental conditions.

Our study has several limitations. First is the limited area of distribution and the limited number of patients pooled from several alpine resorts offering winter sports and mountain biking. The main injuries affected both the upper and lower extremities, as expected, as we treated a limited range of injuries occurring in alpine sports such as skiing, mountain biking and backcountry hiking. Although our findings were consistent in our study, the majority of patients were relatively young to middle-aged, with few pre-existing illnesses. Compared to the cohort in the military studies [8], our median age is higher, thus adding a further degree of general use in a wider age group for trauma analgesia. Second, we did not have complete descriptions of the exact mechanism of trauma in all patients, therefore a correlation between severity of injury (e.g., statistically both a sprained ankle and an open femur fracture affecting the lower extremity) and the expected lower reduction of pain could not be reliably established. Third, interactions between victims and rescuers may have been influenced by sympathy, situation, and temperature, contributing to the victim’s subjective perceptions of pain and pain relief documented by the EMS provider.

## Conclusions

Prehospital OTFC administered in a hostile alpine environment is safe, easy, and efficient for different types of injuries, means of extrication and transport in all age groups and gender; side effects were few and mild. This could provide a valuable alternative without the delay of an intravenous line in trauma patients with severe pain, especially in remote areas, where fast action and easy administration are important.
